# A third generation vaccine for human visceral leishmaniasis and post kala azar dermal leishmaniasis: First-in-human trial of ChAd63-KH

**DOI:** 10.1371/journal.pntd.0005527

**Published:** 2017-05-12

**Authors:** Mohamed Osman, Anoop Mistry, Ada Keding, Rhian Gabe, Elizabeth Cook, Sarah Forrester, Rebecca Wiggins, Stefania Di Marco, Stefano Colloca, Loredana Siani, Riccardo Cortese, Deborah F. Smith, Toni Aebischer, Paul M. Kaye, Charles J. Lacey

**Affiliations:** 1 Centre for Immunology and Infection, Dept. of Biology and Hull York Medical School, University of York, Heslington, York, United Kingdom; 2 Department of Health Sciences, University of York, Heslington, York, United Kingdom; 3 ReiThera Srl (formerly Okairos Srl), Rome, Italy; 4 Keires AG, Bäumleingasse 18, Basel, Switzerland; 5 Agents of Mycoses, Parasitoses and Mycobacterioses, Robert Koch-Institute, Berlin, Germany; University of Notre Dame, UNITED STATES

## Abstract

**Background:**

Visceral leishmaniasis (VL or kala azar) is the most serious form of human leishmaniasis, responsible for over 20,000 deaths annually, and post kala azar dermal leishmaniasis (PKDL) is a stigmatizing skin condition that often occurs in patients after successful treatment for VL. Lack of effective or appropriately targeted cell mediated immunity, including CD8^+^ T cell responses, underlies the progression of VL and progression to PKDL, and can limit the therapeutic efficacy of anti-leishmanial drugs. Hence, in addition to the need for prophylactic vaccines against leishmaniasis, the development of therapeutic vaccines for use alone or in combined immuno-chemotherapy has been identified as an unmet clinical need. Here, we report the first clinical trial of a third-generation leishmaniasis vaccine, developed intentionally to induce *Leishmania*-specific CD8^+^ T cells.

**Methods:**

We conducted a first-in-human dose escalation Phase I trial in 20 healthy volunteers to assess the safety, tolerability and immunogenicity of a prime-only adenoviral vaccine for human VL and PKDL. ChAd63-KH is a replication defective simian adenovirus expressing a novel synthetic gene (KH) encoding two *Leishmania* proteins KMP-11 and HASPB. Uniquely, the latter was engineered to reflect repeat domain polymorphisms and arrangements identified from clinical isolates. We monitored innate immune responses by whole blood RNA-Seq and antigen specific CD8^+^ T cell responses by IFNγ ELISPOT and intracellular flow cytometry.

**Findings:**

ChAd63-KH was safe at intramuscular doses of 1x10^10^ and 7.5x10^10^ vp. Whole blood transcriptomic profiling indicated that ChAd63-KH induced innate immune responses characterized by an interferon signature and the presence of activated dendritic cells. Broad and quantitatively robust CD8^+^ T cell responses were induced by vaccination in 100% (20/20) of vaccinated subjects.

**Conclusion:**

The results of this study support the further development of ChAd63-KH as a novel third generation vaccine for VL and PKDL.

**Trial registration:**

This clinical trial (LEISH1) was registered at EudraCT (2012-005596-14) and ISRCTN (07766359).

## Author summary

Leishmaniasis is a neglected disease of poverty with global public health impact. Caused by species of the *Leishmania* parasite, it may manifest itself as slow to heal skin ulcers, metastatic disease affecting the mouth and nose or systemic disease affecting internal organs (kala azar or visceral leishmaniasis). In common with other poverty-related neglected diseases, there have been few incentives for pharma to develop new drugs and vaccines. The few drugs currently available may cause serious side effects and/or only work well in some settings. No vaccines are available for prevention (prophylactic vaccines) or treatment (therapeutic vaccines). The clinical development of vaccines requires a series of clinical trials (Phases I–IV). Phase I studies assess primarily safety and are conducted in healthy volunteers. Later phase trials then involve either more healthy subjects (for prophylactic vaccines) or patients (for therapeutic vaccines) and provide additional safety data as well as preliminary data on efficacy. Here, we describe the results from a Phase I first-in-human trial of a new vaccine for leishmaniasis. Our vaccine is novel compared to those previously tested in that it is designed to target induction of a specific type of immune response (CD8^+^ T cells). We show here that the vaccine is safe in healthy volunteers and induces robust CD8^+^ T cell responses. The study represents an important step towards the development of a leishmaniasis vaccine.

## Introduction

The leishmaniases represent a group of heterogeneous diseases caused by intracellular protozoan parasites of the genus *Leishmania*. Transmitted by phlebotomine flies, approximately 1.5 million new cases occur each year, across 98 countries worldwide, with 20,000–40,000 deaths [1]. Clinical, epidemiological and experimental evidence suggests that these should be vaccine-preventable diseases: healing of cutaneous leishmaniasis (CL) results in resistance to reinfection; sub clinical infection is common, due to effective cellular immunity; ‘leishmanisation’ was highly successful in protecting against CL; and prior history of CL provides cross protection against visceral leishmaniasis (VL) [2–4]. Furthermore, experimental and clinical data support the development of immuno-chemotherapy and therapeutic vaccination as a future therapeutic option [5–14]. Nevertheless, no vaccines are currently approved for human use. Although prophylactic vaccines for leishmaniasis represent an ultimate goal and are likely to have the widest impact on health, the development path for such vaccines is complex. In the absence of human challenge models or established correlates of protection, a demonstration of protective efficacy necessitates large sample sizes and protracted time scales. Furthermore, the induction of memory T cell responses often requires complex prime-boost schedules. In contrast, a therapeutic vaccine for use in VL or PKDL patients, as a tool to limit VL progression to PKDL and /or to reduce infectiousness of PKDL patients and asymptomatic carriers would have significant benefits both for individuals and communities. The clinical path for development of therapeutic vaccines is considerably more straightforward, with shortened time frames and sample sizes to demonstrate efficacy and a lesser need to establish memory responses [12].

To combat established infection, therapeutic vaccines must overcome parasite survival strategies that subvert either intrinsic macrophage function [15] or extrinsic immune regulatory circuits, such as Th1:Th2 bias, Treg activation, manipulation of checkpoint inhibition and altered host cellular metabolism [9, 16–19]. CD8^+^ T cells play a significant role in all forms of leishmaniasis. As is the case for CD4^+^ T cells, CD8^+^ T cells are mostly host protective but may also drive pathology, depending on the form of leishmaniasis and disease staging [20, 21]. In VL and PKDL, the weight of evidence suggests a host protective role for CD8^+^ T cells, including: studies of adoptive CD8^+^ T cell immunotherapy [22]; the correlation of vaccine-induced immunity with CD8^+^ T cell effector function [12, 23–28]; and the identification of CD8^+^ T cell anergy and / or exhaustion in PKDL patients [29, 30]. Importantly, the therapeutic benefit of overcoming CD8^+^ T cell anergy in pre-clinical models of VL has also been demonstrated [18].

To date, first and second generation vaccines for leishmaniasis have been developed to induce primarily CD4^+^ T cell responses [31, 32]. Here, we report on a first-in-human clinical trial of a novel adenoviral-based vaccine for VL / PKDL, specifically designed to elicit CD8^+^ T cell responses. The vaccine employs a well-tested simian adenovirus, ChAd63 [33–36], encoding a synthetic gene for the co-expression of two *Leishmania* antigens with demonstrated vaccine efficacy in pre-clinical models (KMP-11 and HASPB). Representing a novel approach, a synthetic *haspb* gene was designed to reflect repeat diversity and repeat domain structure of the gene product as known from clinical isolates of *L*. *donovani* from India and East Africa [12]. We show that this vaccine is safe and induces cytokine-producing CD8^+^ T cells in high number and with broad epitope coverage, reflective of an innate immune response involving activated dendritic cells. These data pave the way for evaluating this vaccine for potential therapeutic benefit in PKDL patients, for the prevention of PKDL and in asymptomatic carriers of *L*. *donovani* infection.

## Materials and methods

### Subjects

There was one study group consisting of twenty healthy male and female volunteers aged 18 to 50 years who were willing and able to adhere to the conditions of the trial and to give written informed consent, and who fulfilled the entry criteria. All subjects were negative for rk39.

### Ethical and regulatory approval

The study, designated as LEISH1 (EudraCT 2012-005596-14; ISRCTN 07766359), was approved by the UK National Health Service Research Ethics Committee (North East -York; 13/NE/0071), and the University of York Department of Biology Ethics Committee. LEISH1 was co-sponsored by the University of York and the York Teaching Hospital NHS Foundation Trust (YOR-A01161).

### Study design

LEISH1 was an open label phase I study to assess the safety and immunogenicity of a candidate *Leishmania* vaccine in healthy volunteers. There was no blinding or randomisation or control arm. Subjects were allocated to receive either low dose (1x10^10^ vp; n = 5, 4F and 1M) or high dose (7.5x10^10^ vp; n = 15, 9F and 6M) of ChAd63-KH as a single intramuscular injection. The dose selection for this study was based on existing safety and immunogenicity data for other ChAd-vectored vaccines that indicate a similar safety profile between 10^9^−10^10^ vp, but with increasing immunogenicity [33, 35, 37, 38]. 7.5x10^10^ was selected as the high dose for this study, as increased reactogenicity of ChAd vaccines has been observed above this dose and this was also the maximal dose achievable in a 1ml injection with the vaccine lot produced at GMP. Vaccinations were performed in a step-wise manner, with safety reviews 24h after the first subject received low dose vaccination, after all low dose subjects had attended their day 14 follow up and 24h after the first high dose subject was vaccinated. No more than two subjects were vaccinated on any given day and no subjects were vaccinated simultaneously.

### Vaccine

The ChAd63-KH vaccine is a replication defective simian adenoviral vector expressing KH, a self-cleaving polyprotein comprising *L*. *donovani* KMP-11 and HASPB [12]. The vaccine is presented in glass vials, each vial containing a concentration of 7.5x10^10^ vp / mL (6.5x10^8^ ifu/ml) formulated in buffer A438 (10mM Histidine, 7.5% sucrose, 35mM NaCl, 1mM MgCl_2_, 0.1% PS80, 0.1mM EDTA Disodium, 0.5% ethanol, pH 6.6). Manufacture and labeling of the drug product were carried out in accordance with the requirements of GMP by Advent Srl., Italy.

### Clinical follow up

Subjects were followed up at days 1, 14, 28, 56 and 90 post-vaccination. Adverse events were collected through diary cards, direct questioning, physical examination, and laboratory safety tests.

### Immunogenicity assays

Ex vivo (18h stimulation) assays of frozen and fresh PBMC were performed using Multiscreen IP ELISPOT plates (Millipore), human IFNγ SA-APL antibody kits (Mabtech) and BCIP NBT-plus chromogenic substrate (Moss Inc). Cells were cultured in RPMI (Sigma) containing 10% heat-inactivated, sterile-filtered calf serum (Labtech International). Antigens were tested in duplicate with 250,000 PBMC added to each well of the ex vivo ELISPOT plate. 444 KH peptide sets (each pepset containing an 11mer with its truncated 10mer, 9mer and 8mer, and with each 11mer overlapping by 10 amino acids) were assayed in six pools comprising 105 (pool p1), 57 (pool p2), 81(pool p3.1), 81(pool p3.2), 82 (pool p3.3) and 38 (pool p4) pepsets at 10 μg/ml. A second set of KH peptides of 15 amino acids in length, overlapping by 11 amino acids was also used in three pools of 36 (pool pA), 36 (pool pB) and 38 (pool pC) at 10 μg/ml. Responses were averaged across duplicates, responses in unstimulated (negative control) wells were subtracted and responses in individual pools were summed across the KH antigen, as indicated. Staphylococcal enzyme B (Sigma) at 0.04 μg/ml was used as a positive control. Plates were counted using an AID automated ELISPOT counter (AID Diagnostika, GmbH, algorithm C), using identical settings for all plates. Responses to negative controls were always <50 SFC per million PBMC. Responses of >50 SFC per million for single peptide pools after subtraction of background (negative control) were considered positive. Subjects were considered as responders when pooled sum responses were >200 spots per million PBMC. All results presented were derived from batched assays conducted with previously frozen PBMC for greater consistency.

For flow cytometry, responses were assessed by a 7-colour staining panel. Aliquots of 1x10^6^ cells were plated in 96-well plates in 200 μl of medium and stimulated with either no antigen, peptide pools spanning the KH antigen (pools 1–4; 1 μg/ml) or with Staphylococcal enterotoxin B (Sigma, 1 ug/ml) for 18 h. Brefeldin A (Sigma; 1 μg/ml) was added for the last 16 h. Cells were incubated with a live-dead discriminating dye (Viability dye e780, 1/1000 eBioscience), and then surface—stained with anti-CD3 eFluor 450 (1/50, eBiosciences), anti-CD4 FITC (1/20, eBioscience) and anti-CD8 Percp-Cy5 (1/50, eBioscience). After permeabilisation, intracellular staining was performed with anti-IFNγ PE-Cy7 (1/50), anti-TNF APC (1/50) and anti-IL-2 PE (1/50, all eBioscience) and fixed in 4% paraformaldehyde. Acquisition was performed on the day of staining on a CyAn ADP (Beckman Coulter) and at least 500,000 events were collected per sample. Data were prepared and analysis performed using FlowJo 7.6.5 (Treestar Inc.). Cells were gated on lymphocytes, singlets, live CD3^+^, CD8^+^, and then cytokine combinations (IFNγ, TNF and IL-2). Responses to peptide were determined after subtraction of the response in the unstimulated control for each sample. Subjects were classed as responders to individual peptide pools when response exceeded 0.05% of CD8^+^ T cells.

Anti-KH IgG ELISAs were performed in Nunc-Immuno Maxisorp 96-well plates (Thermo Scientific) coated with 1 μg/ml of KH protein in carbonate-bicarbonate coating buffer (Sigma) overnight at 4 ^o^C. Plates were washed with PBS Tween and blocked with 1% BSA. Sera were diluted at starting concentration of 1:100, added in duplicate and then serially diluted. Plates were incubated for 2 h at room temperature and then washed as before. Goat anti-human whole IgG conjugated alkaline phosphatase (Sigma) was added for 1 h at room temperature. After a final wash, plates were developed by adding p-nitrophenylphosphate at 1 mg/ml in diethanolamine buffer (Peirce). Optical density (OD) was read at 405 nm on an ELx800 microplate reader and data are shown after subtraction of day zero readings. Subjects were considered responders with an OD>0.1 on at least one time point.

### Whole blood transcriptomics

Whole blood samples pre and post vaccination were collected into PAXGene tubes and frozen at -80. Data generation and analysis were carried out by the Centre for Genomic Research, based at the University of Liverpool to provide DE gene lists (see **S**1 **Text** for further details). The reference genome used for alignment was the human reference genome assembly GRCh37/hg19. R1/R2 read pairs were mapped to the reference sequence using TopHat2 version 2.0.10 which calls the mapper Bowtie2 version 2.1.0. Paired-end mapping was carried out using default parameters except for the option to report a maximum of 1 alignment to the reference for each read, instead choosing the alignment with the best alignment score (or randomly choosing among equally high scoring alignments) (option “-g 1”). Read counts per gene were calculated using HTSeq-count (http://wwwhuber.embl.de/users/anders/HTSeq/doc/count.html). Differential gene expression (DGE) analysis was applied to the read count data for reads mapped to the human genome.

The analysis was conducted in the R environment using edgeR. For subsequent deconvolution of the data and identification of major leucocyte subsets, we inputted FPKM values into CIBERSORT [39]. Data were further explored using Ingenuity Pathway Analysis (Qiagen, redwood City, CA, USA) and gene set enrichment analysis (GSEA) using tools developed at the Broad Institute (MIT, Boston, USA).

## Results

### Study population

**Fig 1:**
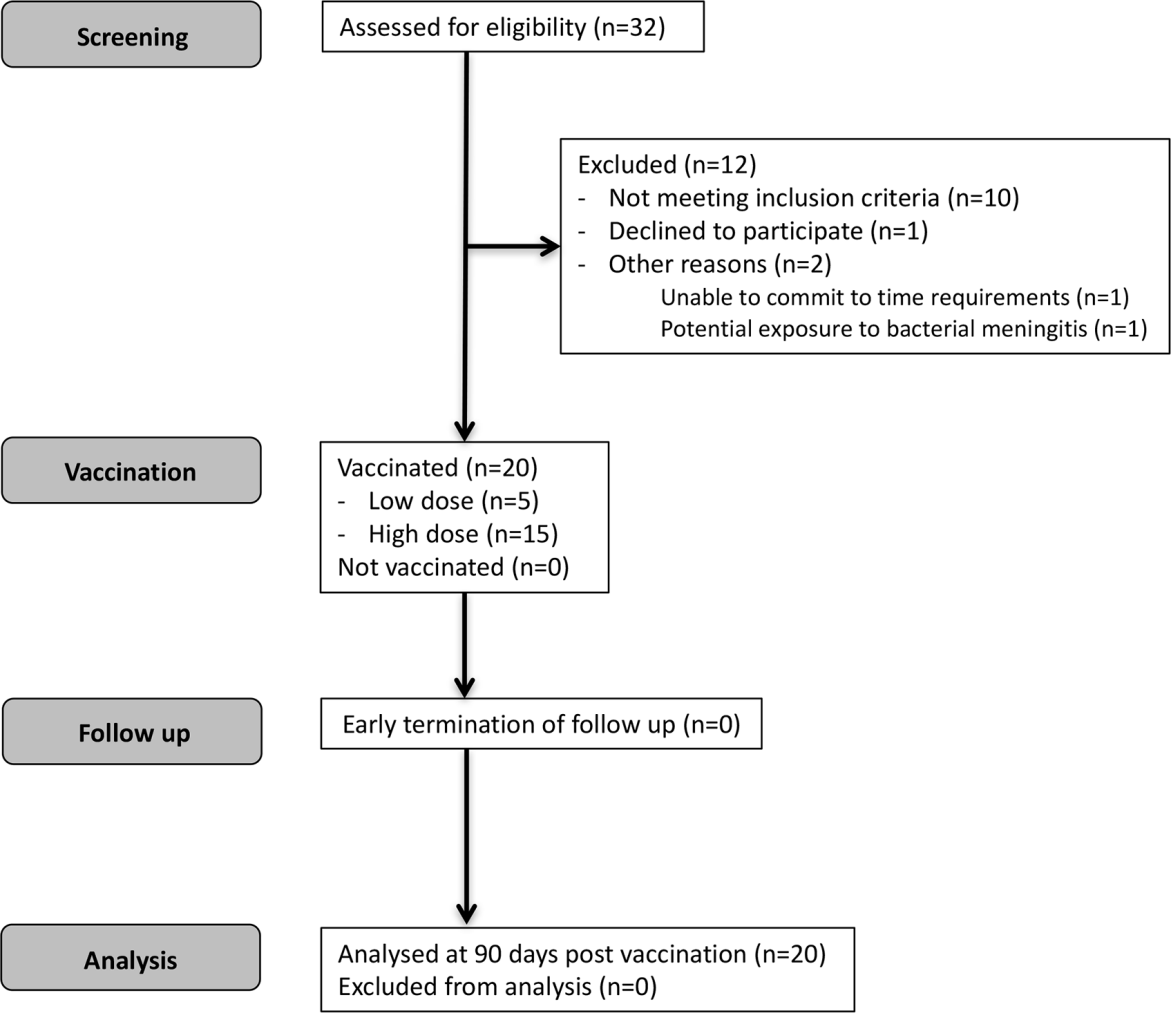
CONSORT diagram for LEISH1 first-in-human clinical trial.

32 individuals were screened for eligibility (**Fig 1**) and 20 subjects with the demographic characteristics shown in **Table 1** were enrolled, vaccinated and followed up. All subjects completed the study to day 90 post vaccination.

**Table 1:** Study population.

Characteristic*	Low dose (n = 5)	High dose (n = 15)
**Gender**		
Male (%)	1 (20%)	6 (40%)
Female (%)	4 (80%)	9 (60%)
**Age**		
N (%)	5 (100%)	15 (100%)
Mean (SD)	35.8 (10.57)	28.7 (8.28)
Median (min, max)	41 (20, 45)	31 (19, 44)
IQR [25%, 75%]	[30, 43]	[20, 36]
**BMI**		
N (%)	5 (100%)	15 (100%)
Mean (SD)	25.0 (2.00)	24.3 (3.43)
Median (min, max)	24 (23, 28)	23 (19, 30)
IQR [25%, 75%]	[24, 26]	[21, 27]

*HLA typing data is provided in **S5 Table.**

### Safety

**Fig 2:**
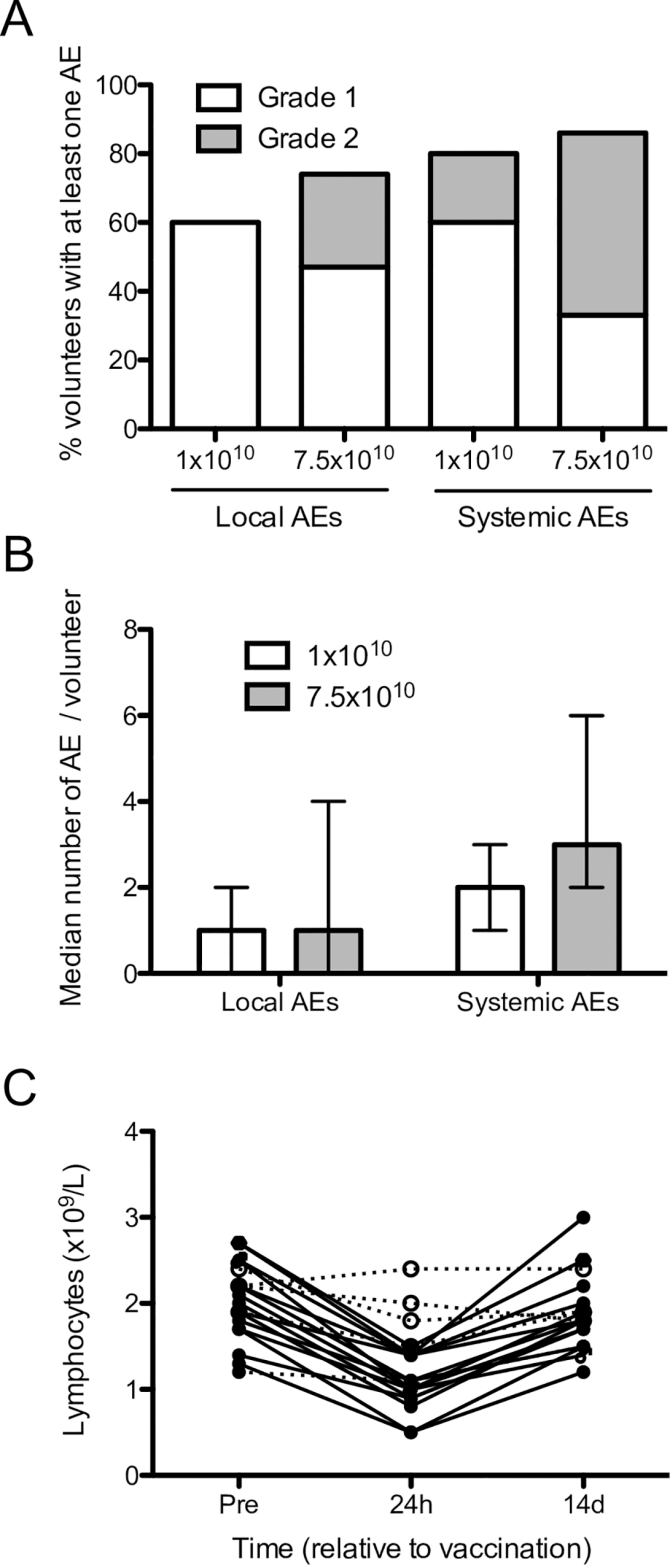
Summary of local and systemic adverse events in LEISH1. **A.** Percentage of subjects vaccinated with low or high dose ChAd63-KH with at least one grade 1 (open bar) or grade 2 (grey bar) adverse events. **B.** Number of local and systemic adverse effects in subjects vaccinated i.m. with 1x10^10^ vp (open bar) or 7.5x10^10^ vp (grey bar). Data are shown as median ± interquartile range. **C**. Individual peripheral blood lymphocyte counts in low dose (open circles) and high dose (black circles) subjects pre- vaccination and at 24h and 14 days post-vaccination. Significant lymphopenia was defined as >25% reduction in lymphocyte count.

Local and systemic AEs were limited to Grades 1 and 2, and none were categorized as serious. There were no SUSARs or SAEs reported in this trial. Three of five (60%) subjects had at least one local AE in the low dose group, compared to 11 of 15 (73%) in the high dose group (**Fig 2A**). In total, 35 local adverse events (median = 1 event per patient) were reported across all subjects. These were largely injection site reactions. Twenty-five events (71%) were related to the vaccinated arm, and only these events were defined as at least possibly related to vaccination. In addition, 4 of 5 (80%) subjects had at least one systemic AE in the low dose group, compared to 13 of 15 (87%) in the high dose subjects (**Fig 2A**). In total, 64 systemic adverse events (median = 2 events per subject with low dose and 3 events per subject with high dose) were reported, 28 of which (44%) were defined as at least possibly related to vaccination (**Fig 2B**). Overall, AEs were not significantly different between the two doses and were similar to those reported for other ChAd63 vaccines [33–36]. A transient lymphopenia, as expected [33, 37, 38, 40–43], was observed in 15/15 high dose-vaccinated subjects (p<0.001) and 0/5 low dose-vaccinated subjects (p = 0.18; **Fig 2C**). Overall, these data indicate that vaccination with ChAd63-KH is safe.

### Whole blood transcriptional response to ChAd63-KH vaccine

**Fig 3:**
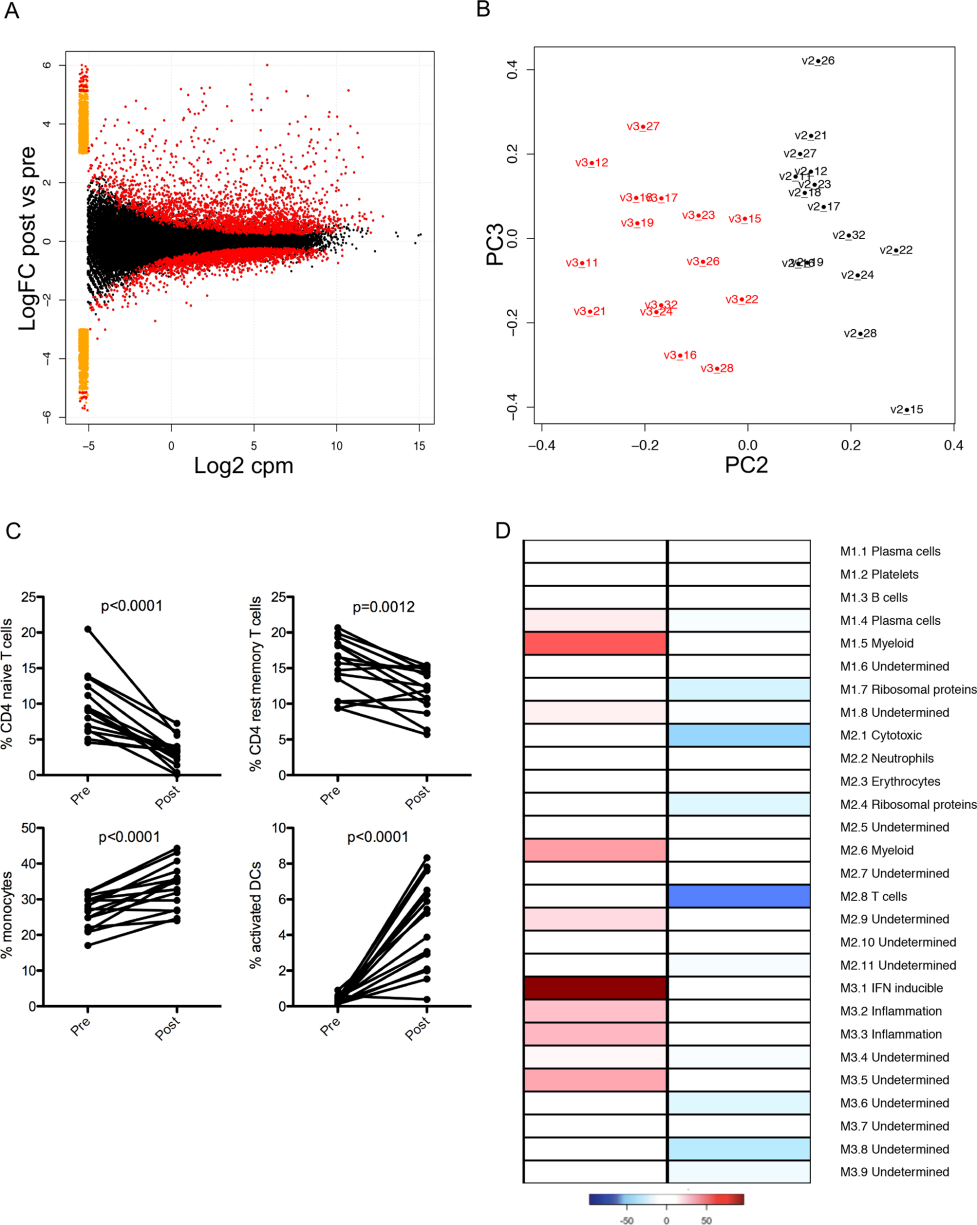
Innate immune response to ChAd63-KH vaccination. Whole blood from high dose subjects was collected before vaccination and at 24h post vaccination and processed for RNA-Seq. **A.** Volcano plot showing Log2FC in gene expression (y axis) against signal intensity (Log2CPM). **B.** Principle component analysis showing clustering of pre- (black, by subject number) and post- (red, by subject number) vaccination samples. **C.** Frequency of naïve and resting memory CD4^+^ T cells, monocytes and activated DCs pre and post vaccination, as determined by CIBERSORT analysis. **D**. Module level analysis comparing gene representation pre and post vaccination. Colour code represents proportion of genes significantly changed (over-represented, red; under-represented, blue) for each of the 28 modules described by Chaussabel et al [47].

We used RNA-Seq to profile the whole blood transcriptome at 24h post vaccination, allowing comparison of the innate response induced by ChAd63-KH with other human and murine studies [44–46]. Analysis of high dose subjects revealed 4799 transcripts that were differentially represented (denoted as differentially expressed, DE) in whole blood (FDR 0.05; 2542 UP; 2257 DOWN; **Fig 3A and S1 Table**), with a clear distinction between pre- and post-vaccination samples (**Fig 3B**). In contrast, analysis of the five low dose subjects identified only 122 DE transcripts (107 UP, 15 DOWN) (**S2 Table**), of which 103/122 (84.4%) were also DE in high dose subjects.

Given the lymphopenia observed in high dose subjects, we used CIBERSORT ([39] to computationally resolve cell subset composition from the transcriptomic data. In low dose subjects, no significant changes were observed between pre- and post-vaccination samples for any of the leucocyte subsets evaluated. High dose vaccination, however, resulted in a significant reduction in the frequency of naïve and resting memory CD4^+^ T cells, with respective increases in the frequency of monocytes and activated DCs (**Fig 3C and S1 Fig)**. We next scored the frequency of transcripts that were significantly changed in high dose subjects for the 28 immune-related modules described by Chaussabel et al [47] **(Fig 3D**). Transcripts contained within modules that related to myeloid cells (M1.5, M2.6), to interferon inducible genes (M3.1) and that were associated with inflammation (M3.2, M3.3) were over-expressed in post vaccination samples. In contrast, modules related to T cells (M2.1, M2.8) and ribosomal protein genes (M1.7, M2.4) were under-expressed. Finally, we conducted a gene set enrichment analysis using the modules described by Li et al [48]. Post vaccination samples were significantly enriched for modules related to viral sensing and monocyte / DC activation, whereas pre- vaccination samples were enriched predominantly for modules defining T cells (**S3 Table**). Collectively, these data point to a lymphopenia predominantly affecting CD4^+^ T cells, and provide evidence of DC activation as an early consequence of innate immune activation.

**Fig 4:**
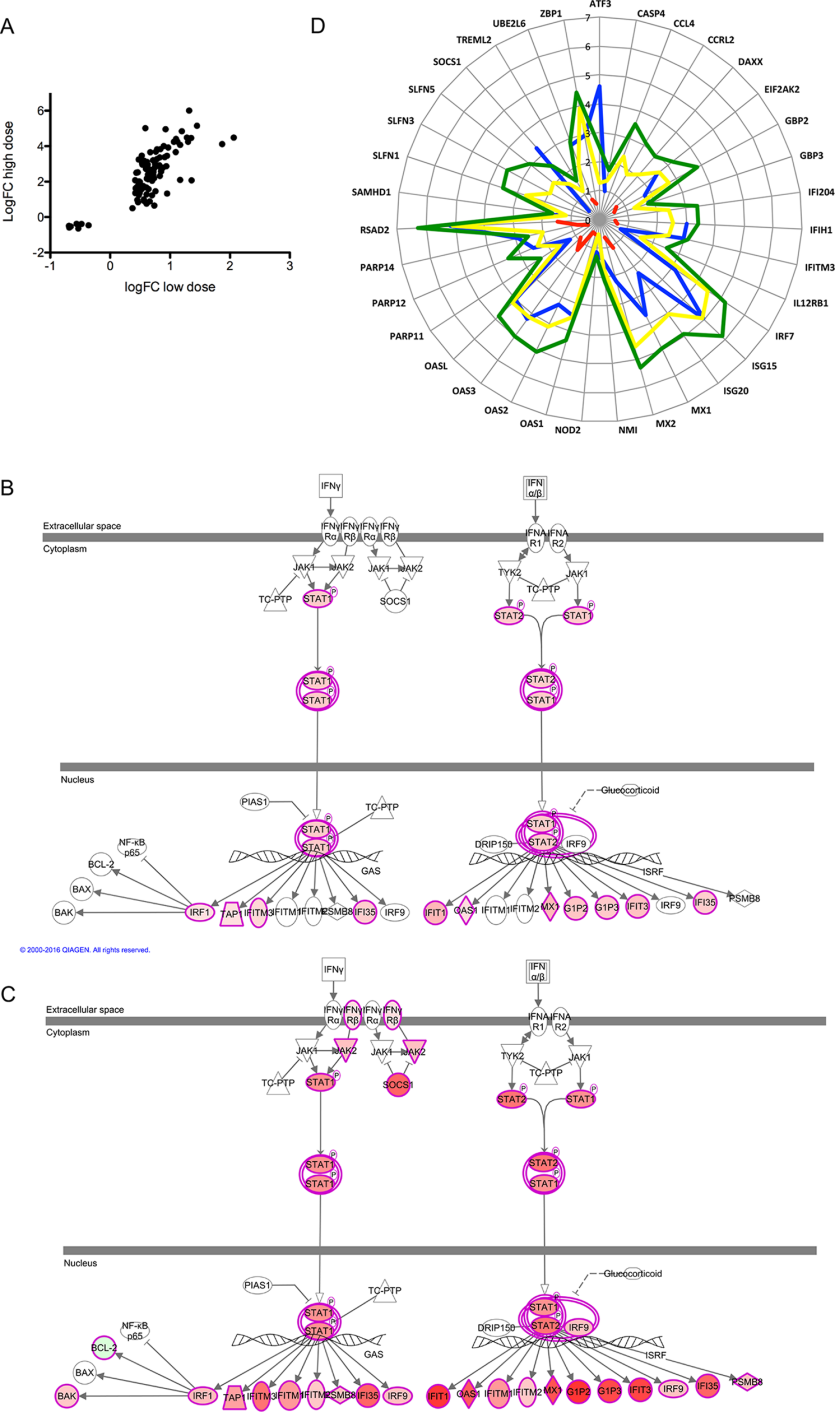
Interferon signatures associated with ChAd63-KH vaccination. **A.** Scatter plot showing magnitude of differential expression for the 103 genes commonly regulated in low and high dose subjects. **B and C.** DE genes associated with the IPA canonical IFN pathway in low dose (B) and high dose (C) subjects. Degree of fold change is shown in red scaling. **D**. Radar plot showing fold change in gene expression for genes identified by Quinn et al as related to antigen presentation (module C2; [44]). Data are shown for low dose (red) and high dose (blue) subjects, as well as for mice immunized with ChAd3 (yellow) and ChAd63 (green). Mouse data originated from Quinn et al [44].

Next, we analyzed DE genes using Ingenuity Pathway Analysis, identifying positively scoring canonical pathways associated with phagocyte and APC function and negatively scoring pathways associated with T cell activation (**S2A Fig**). Key regulators upstream of myeloid cell differentiation and function included CORT, FANCA, LGR4, PLA2G2D, PLA2G10, DEPTOR and RNASE2 (**S2B Fig**). 100/103 (97%) of the commonly DE transcripts showed a greater fold change in high dose subjects than in low dose subjects (**Fig 4A**), likely reflecting the effects of both monocyte/DC enrichment and vaccine dose response. An interferon signature was prominent in both high and low dose subjects (**Fig 4B and 4C**), with IFNG, IRF7, IFNL1, IFNA2, STAT1 and IRF3 amongst the most highly IPA-predicted upstream regulators (**S4 Table**).

Finally, Quinn et al recently reported that antigen expression by recombinant viruses was negatively correlated with their ability to trigger activation of selected immune response genes (Module C2; [44]). We found that with few exceptions (ATF3, SOCS1), the response to immunization of humans with 7.5 x 10^10^ vp of ChAd63-KH was broadly similar to that seen in mice immunized with ChAd vectors. However, few Module C2 genes were DE following low dose immunization (**Fig 4D**).

### CD8^+^ T cell responses induced by vaccination

**Fig 5:**
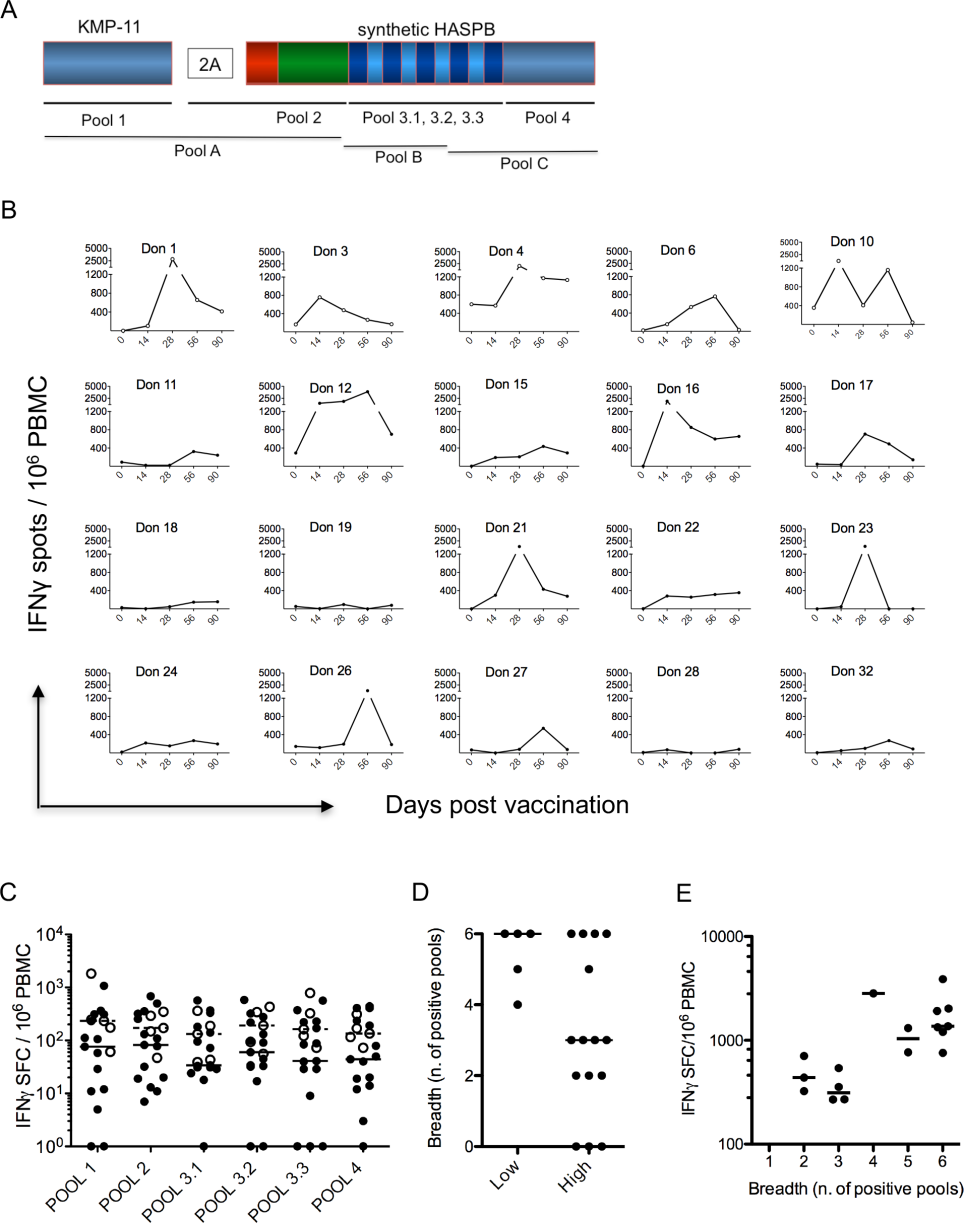
CD8^+^ T cell responses to ChAd63-KH vaccination. **A.** Schematic to illustrate coverage of the KH antigen by the CD8 selective peptide pools (Pools 1, 2, 3.1, 3.2, 3.3 and 4) and CD4/8 15mer peptide pools (Pools A, B and C) used in this study. **B.** ELISPOT response to ChAd63-KH vaccination over time by individual low dose (Don 1, 3, 4, 6, 10; open symbols) and high dose (closed symbols) subjects. Data represent sum of response to all peptide pools at each time indicated. Average responses for low and high dose subjects are shown in S3 Fig. **C.** Peak response to each peptide pool for low (open circles) and high (black circles) dose subjects. Median responses for high (solid line) and low (dotted line) dose subjects are also shown. **D**. Breadth of response, reflecting number of peptide pools recognized by each subject group. **E**. Correlation between breadth of response and sum of total response (R^2^ = 0.3437, p = 0.0134).

To assess immunogenicity, we focused primarily on the induction of effector CD8^+^ T cell responses, determined by direct IFNγ ELISPOT using a pools of pepsets, each containing 8–11 amino acid peptides [12] that span the KH vaccine antigen (**Fig 5A**). All results presented here were obtained from assays conducted with previously frozen PBMC for greater inter-assay consistency. Analysis of the response across all peptide pools on an individual basis (**Fig 5B)** and collectively (**S3 Fig**) indicated that: i) there was an overall response rate of 17/20 (85%), with 5/5 (100%) responders in the low dose vaccine group (subjects 1, 3, 4, 6, and 10) and 12/15 (80%) responders in the high dose group (using a non-responder cut off of <200 spots); ii) for most subjects, the response showed the characteristic expansion and contraction phases of a peripheral T cell immune response; iii) in 8/20 (40%) subjects, peak summed responses across all pools were greater than 1200 spots per million PBMC (low dose: mean 1537, 95% CI 447, 2627; high dose: mean 866, 95% CI 308, 1424; p = 0.08); and iv) time to peak response varied from 14–56 days, but with some responders showing a relatively flat kinetic. The median number of pools recognized by low dose subjects was six, greater than that recognized by high dose subjects which was three (p = 0.043; **Fig 5D**) and there was a significant correlation between number of pools recognized and total IFNγ response (**Fig 5E**; R^2^ = 0.3437, p = 0.0134). However, we found no significant correlations between the summed peak ELISPOT response to peptide pools p1-4 in high dose subjects and: i) the extent of lymphopenia; ii) the respective changes in whole blood leucocyte subset frequency; or iii) the relative enrichment for different gene expression modules.

**Fig 6:**
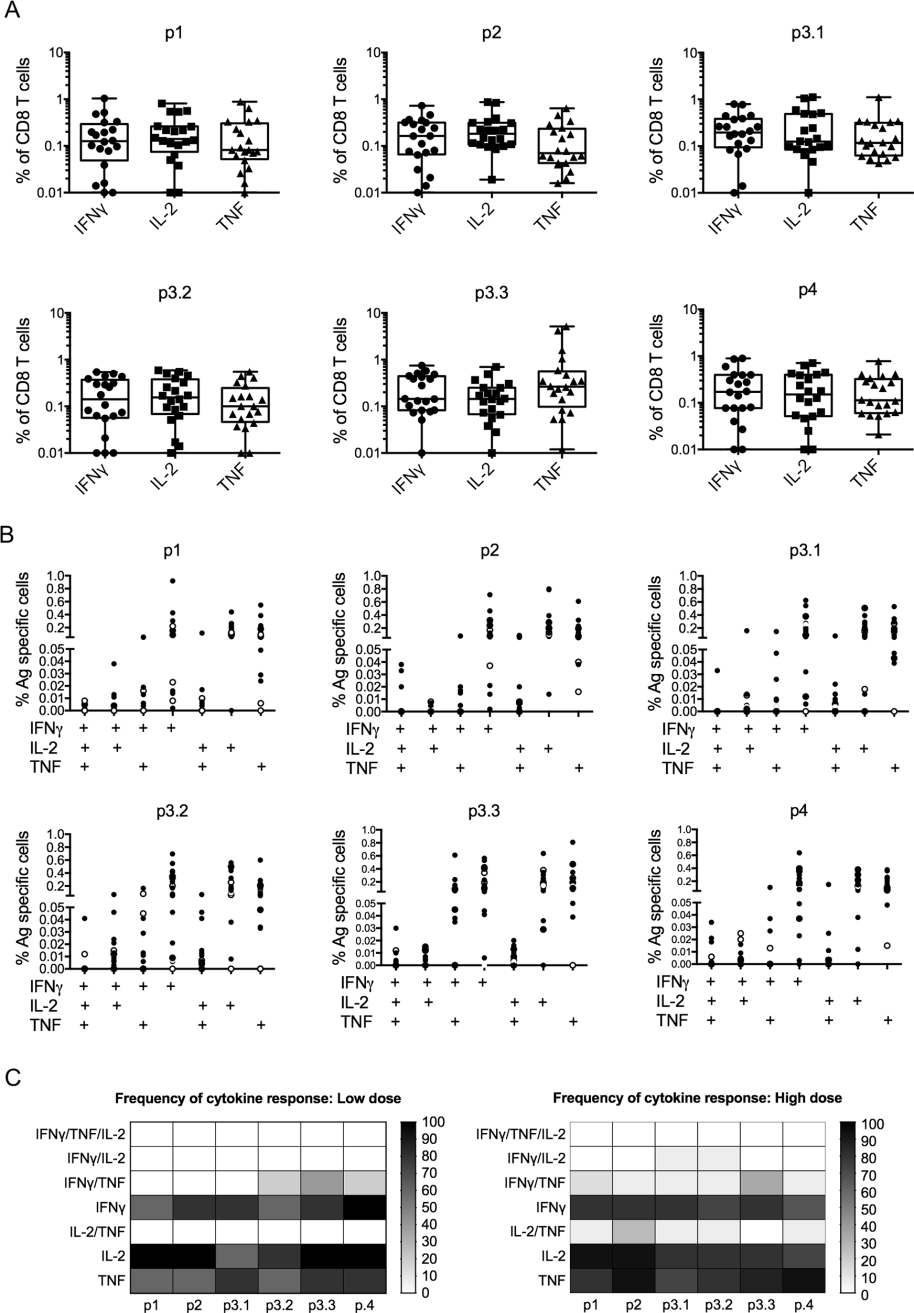
Intracellular cytokine production by CD8^+^ T cells. **A.** IFNγ, TNF and IL-2 responses at day 28 post vaccination to individual peptide pools. Box and whisker plots showing frequency of cytokine producing cells as % of total CD8^+^ T cells. Data are pooled for all low and high dose subjects (n = 20). **B.** Cytokine producing CD8^+^ T cells producing one, two or three cytokines are shown by individual donor for each peptide pool. Low dose (open circles) and high dose (closed circles) subjects are shown separately. **C.** Heat map to show frequency of low dose (left; n = 5) and high dose (right; n = 15) subjects responding (at a cut-off of 0.05%) with single, dual or triple cytokine production after stimulation with each peptide pool.

We next analyzed cytokine production by CD8^+^ T cells using intracellular cytokine staining (ICS), limiting our analysis to cells obtained at d28 post vaccination, a time-point when most subjects responded by ELISPOT. CD8^+^ T cells readily produced IFNγ, TNF and IL-2 in response to peptide stimulation (**Fig 6A**). Analysis of co-expression of IFNγ, TNF and IL-2 across all subjects indicated that responses primed with ChAd63-KH in naïve individuals were dominated by single cytokine producing CD8^+^ T cells (**Fig 6B**). Dual cytokine-producing cells were observed in all combinations, but triple cytokine-producing cells were rarely detected (**Fig 6A and 6C**). Analysis by individual peptide pool and subject revealed a cytokine response to at least one peptide pool in 5/5 of low dose subjects and 15/15 of high dose subjects, with no evidence of immune-dominance or cytokine selectivity associated with any of the peptide pools (**Fig 6C**). Of note, the three high dose subjects previously scored as non-responders by IFNγ ELISPOT (subjects 18, 19 and 28) made cytokine responses when assayed by ICS (**S4 Fig**), indicating an overall CD8^+^ T cell response rate following vaccination of 100%.

### CD4^+^ T cell and antibody response to vaccination

**Fig 7:**
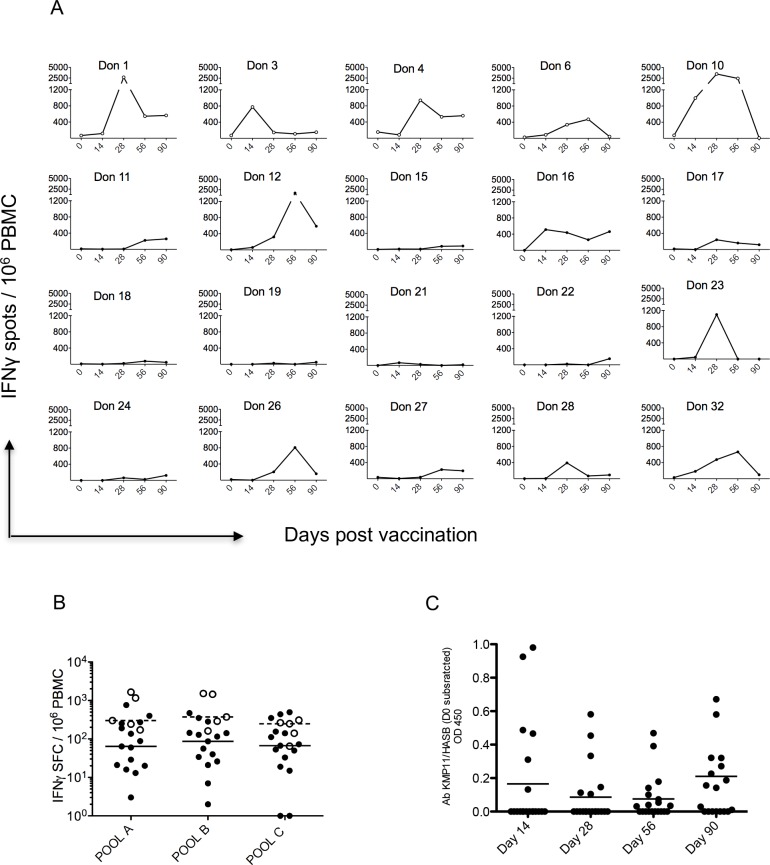
Antibody and CD4^+^/CD8^+^ T cell responses to ChAd63-KH vaccination. **A.** ELISPOT response to ChAd63-KH vaccination over time for individual low dose (Don 1, 3,4,6,10, open symbols) and high dose (closed symbols) subjects. Data represent sum of response to peptide pools A, B and C at each time indicated. **B**. Peak response to each peptide pool for low (open circles) and high (black circles) dose subjects. Median responses for high (solid line) and low (dotted line) dose subjects are also shown. **C.** Antibodies specific for the rKH protein were assayed by ELISA. Data are shown for all subjects at the indicated times post vaccination.

To include potential CD4^+^ T cells responses (as well as CD8^+^ T cell responses), we restimulated PBMC using an alternate set of 15mer peptides (pools A, B and C) (**Fig 5A**). The overall responder frequency was 70% (14/20; **Fig 7A**), possibly reflecting less efficient antigen processing. Nevertheless, responses to these peptide pools were also robust **(Fig 7B)**, again with a suggestion of greater peak summed responses in low dose subjects (low dose: mean 1668, 95% CI 19, 3317; high dose: mean 413, 95% CI 153, 673; p = 0.015). One subject (number 28) responded to these pools but not to the shorter truncated peptide sets used to specifically measure CD8^+^ responses.

Measurement of IgG responses by ELISA against recombinant KH protein demonstrated that ChAd63-KH induced a modest antibody response, measurable on at least one time point, in 3/5 of low dose subjects and 10/15 of high dose subjects (**Fig 7C**). A correlation between antibody response and IFNγ response to pools ABC was only observed at day 90 post vaccination (p = 0.0356; R^2^ = 0.2973). We did not find any significant correlations between antibody responder versus non-responder status with any of the variables (lymphopenia, subset composition or module response) measured at 24h post vaccination.

## Discussion

The development of vaccines for the prevention and treatment of leishmaniasis represents a significant unmet medical need, and although it is well recognised that leishmaniasis provides a good target for vaccination [2], progress towards this goal has been frustratingly slow. Here, we report on the safety and immunogenicity of a novel vaccine designed for induction of CD8^+^ T cells. Although the design of the vaccine is compatible with both prophylactic and therapeutic use, our initial clinical development plan for ChAd63-KH is focused on use of this vaccine as a single dose therapeutic agent. We show that single dose immunization of healthy adults with this simian adenoviral-vectored vaccine, ChAd63-KH, was safe and induced potent CD8^+^ T cell responses to a wide range of epitopes.

First generation prophylactic vaccines for leishmaniasis were shown to be ineffective, in spite of their ability to induce reasonable Th1 type cytokine responses [31]. Nevertheless some of these vaccines have been shown to have promise in a therapeutic setting [5–7]. The range of second-generation recombinant protein-based vaccines investigated in pre-clinical animal models, including mice, hamsters and primates, is constantly growing but few have been pursued through to clinical trial (reviewed in [2, 4]). Recent clinical activity has been largely focused on the improvement of adjuvants for use in combination with recombinant poly-protein vaccines [32, 49–53]. However, until now, no clinical trials have been conducted with leishmaniasis vaccines where delivery route has been specifically selected to induce CD8^+^ T cells, namely third generation DNA and viral vaccines.

The induction of CD8^+^ T cells has been the mainstay of vaccine development for other intracellular pathogens [35, 40, 54, 55], and there is ample evidence to support a role for CD8^+^ T cells in immune mediated protection against leishmaniasis [18, 20, 21, 23, 26, 56–58]. Notably, HuAd5-KH demonstrated therapeutic benefit after a single vaccination in an experimental model of chronic *L*. *donovani* infection [12] and HuAd5-A2 (expressing the *Leishmania* A2 antigen) was shown to have prophylactic benefit in a primate model of infection, albeit requiring a recombinant A2 protein / rIL-12 boost [28]. For clinical use, simian adenoviruses are now widely regarded as one of the most effective means to induce CD8^+^ T cell responses, and ChAd-based vaccines against malaria, TB, HCV, HIV, Ebola, and Rift Valley fever are in clinical development [33, 37, 40, 42, 59]. ChAd63-KH appears to have a similar safety profile to these vaccines, suggesting that from a safety perspective, ChAd63-KH is suitable for further clinical development.

ChAd63-KH has many novel features not previously adopted in the leishmaniasis vaccine development pipeline. First, we co-translationally expressed two leishmanial antigens in the same vector using a viral 2A sequence, saving costs compared to the generation of modular vaccines [26], and obviating the potential of fused polyprotein vaccines to create neo-epitopes and/or alter immunogenicity [60]. 2A sequences have been used in gene therapy applications [61, 62] in experimental vaccines [12, 63], and in an RSV vaccine clinical trial [64], but this is the first time such a strategy has been used in a leishmaniasis vaccine. Second, we engineered the repeat sequences of the *haspb* gene to take account of the sequence diversity and repeat structure found in field isolates of *L*. *donovani* in India and East Africa, the principal Old World endemic settings for VL and PKDL. This is the first time that parasite strain diversity has been taken into account in this way in a leishmaniasis vaccine. It is noteworthy that the repeat regions of HASPB appear well recognized in vaccinated humans, in keeping with their presumptive role in vaccine-induced efficacy in animal models [23, 24].

Although there was considerable heterogeneity of response at the individual level, across all subjects studied, we were not able to assign any significant immuno-dominance to either antigen or to any specific region of HASPB. KMP-11 is non-polymorphic and in vitro mapping of immunogenic peptides of KMP-11 had suggested that this molecule contained a plethora of epitopes available for recognition by multiple class I loci [57]. This was borne out by the high response rate of ChAd63-KH vaccinated subjects to peptide pool 1 (60% by IFNγ ELISPOT; 100% for any cytokine by ICS). Furthermore, in addition to responses targeted to the repeat regions of HASPB, the high level of response to the conserved HASPB N and C termini supports the use of this antigen as a potential vaccine candidate for strains of *L*. *donovani* where there may be additional repeat diversity beyond that captured in our synthetic gene, and for species where similar conserved regions are present in HASP proteins (e.g. *L*. *major and L*. *mexicana*). Hence, our initial immunogenicity data in vaccinated subjects suggests that the KH antigen fulfills the criteria for a pan-leishmaniasis vaccine candidate. Although the combination of KMP-11 and HASPB gives excellent broad epitope recognition in naïve subjects (combined responses rates of 85% for IFNγ ELISPOT; 100% for any cytokine), qualitative or quantitative improvement on this response might still be possible, through alternate immunization schedules (e.g. prime-boost) or the introduction of additional vaccine antigens. The cost of developing vaccines at GMP precludes the separate evaluation of ChAd63-KMP-11 and ChAd63-HASPB as vaccine candidates in humans.

The CD8^+^ T cell response elicited by a single dose vaccination with ChAd63-KH appears dominated by IFNγ, TNF or IL-2-producing cells, with minimal evidence of poly-functionality. This may not be wholly surprising, given that previous clinical studies have shown that poly-functionality becomes more manifest following prime-boost vaccination [42]. Although there is considerable evidence that poly-functionality of the CD4^+^ T cell response correlates with vaccine-induced protection in mouse models of cutaneous leishmaniasis [65, 66], similar data are unavailable from human leishmaniasis vaccine studies, where cytokine profiling has been confined to culture supernatants [53]. Likewise, although we have shown poly-functional responses following therapeutic vaccination with HuAd5-KH in mice [12], direct evidence of their superiority to single cytokine producing T cells in any therapeutic setting is lacking. Of note, single cytokine producing CD8^+^ T cells have been correlated with protection against influenza virus infection [67]. Given the diversity of human leishmaniasis and the variable settings for which a leishmaniasis vaccine may be deployed, correlating cytokine responses at the single cell level with clinical vaccine efficacy data will be required to identify a dominant protective role for polyfunctional T cells.

For the first time in the context of a leishmaniasis vaccine trial, we conducted whole blood transcriptomic profiling of the innate immune response. Not surprisingly, we observed a response at 24h post vaccination dominated by an interferon signature, as noted in similar analyses of other vaccines [44, 48, 68–70]. Transcriptomic analysis using whole blood was confounded by an observed lymphopenia in recipients of high doses of ChAd63-KH, leading to an apparent increase in myeloid cell frequencies and consequently an artificial enrichment of related cell-specific transcripts. Since this is a common confounder, we used leucocyte subset deconvolution to confirm that monocyte and DC activation resulted from vaccination. Although the current study was not designed to detect significant differences in immunogenicity between low and high dose subjects, low dose subjects showed a trend towards generating broader responses. Previous transcriptomic analysis of the murine response to various ChAd vectors identified a module of interferon-stimulated genes (Module C2) that was indicative of heightened innate immunity at the expense of: i) vector-driven antigen expression in dendritic cells; and ii) subsequent CD8^+^ T cells response [44]. Although we could not directly measure antigen expression in this study, it is noteworthy that most Module C2 genes changed in their expression following high dose vaccination with ChAd63-KH, whereas few were changed following low dose vaccination. Hence, it is tempting to speculate that the lower innate response provoked by low dose vaccination may support a broader CD8^+^ T cell response by allowing for heightened antigen expression. Should this be borne out in further studies in patients (see below), low dose vaccination schedules may provide considerable cost savings.

Although we are proposing clinical development of ChAd63-KH first in a therapeutic context, we are aware of potential challenges ahead. For example, therapeutic vaccination with ChAd3-NSmut/MVA-NSmut was unable to overcome the immune dysregulation associated with chronic Hepatitis C Virus (HCV) infection and vaccine-induced T cell responses were impaired compared to that seen in healthy vaccine recipients [71]. Although qualitatively similar immune dysregulation is observed in active VL and to a lesser extent in PKDL [19, 72], experimental data in models of established VL suggest that therapeutic vaccination with viral vectors can nevertheless lead to functional T cell activation and reduced parasite burden [12, 18]. Whether this can be recapitulated in humans with PKDL (for which there is no animal model) or in asymptomatic VL patients (where immune dysregulation may be less evident) remains to be tested in the clinic. New options for boosting the efficacy of therapeutic vaccines are also being developed. For example, recent studies in SIV-infected rhesus monkeys indicate that combining therapeutic vaccination with Ad26/MVA in conjunction with TLR7 stimulation can lead to T cell responses capable of inducing viral clearance [73]. Similar approaches could be tested in leishmaniasis once ChAd63-KH has been shown to be safe through dose escalation and age de-escalation safety studies in patients.

A therapeutic vaccine for VL / PKDL could have major health impacts: preventing PKDL development after treatment for VL; increasing cure rate in PKDL patients (with or without drug therapy); and protecting communities against ongoing VL transmission. Although extensive economic modeling studies to show the cost-benefit of therapeutic vaccines in PKDL has not been performed to date, similar studies for VL vaccines suggest that a prophylactic vaccine would be highly cost effective even at a cost of $100 [74]. In addition to our safety and immunogenicity data, the absence of a requirement for long lasting memory cell induction, the relatively short time scales and small sample sizes involved in assessing therapeutic efficacy and an alignment to established drug target product profiles provide a compelling case for further clinical evaluation of this new treatment strategy.

## Supporting information

S1 Text (DOCX)

S1 Table DE gene list for high dose subjects. (XLS)

S2 Table DE gene list for low dose subjects. (XLSX)

S3 Table Gene set enrichment analysis using modules described by Li et al. (XLSX)

S4 Table IPA predicted upstream regulators in high and low dose subjects. (XLSX)

S5 Table HLA typing of study subjects. (PDF)

S1 Fig Blood leucocyte composition pre- and post-vaccination. **A and B.** High resolution blood composition was inferred by deconvolution of whole blood RNA-Seq data using CIBERSORT. Data are shown for each low dose (A) and high dose (B) subject pre- and post-vaccination. **C.** Differential blood counts obtained by routine clinical hematology for all subjects pre and post vaccination.
(PDF)

S2 Fig Pathway analysis of DE genes. **A.** Top 16 enriched IPA canonical pathways. Bars (left axis) indicate percentage of genes per pathway up-regulated (red) or down-regulated (green). Line graph (right axis) indicates log10 probability (P value) vs. randomly selected gene group of same size. Numbers above bars indicate the number of genes included each pathway. **B.** IPA-derived regulator map for monocyte / macrophage activation.
(PDF)

S3 Fig Kinetics of IFNγ response to vaccination. **A-C.** IFNγ response determined by ELISPOT before and at the indicated times following vaccination of low dose (A and C, left panel) and high dose (B and C, right panel) subjects to individual peptide pools spanning the KH antigen (A, B). The summed response is also shown (C). Data are shown as box and whisper plots.
(PDF)

S4 Fig Cytokine production by KH-specific CD8^+^ T cells. IFNγ (black bars), TNF (grey bars) and IL-2 (white bars) were measured by ICS in CD8^+^ T cells at day 28 post-vaccination for ELISPOT non-responder subjects 18, 19 and 28. Data represent mean frequency of antigen-specific T cells producing each cytokine in response to peptide pools spanning the KH antigen (p1, p2, p3.1, p3.2, p3.3, p4).
(PDF)
