# Supplementary material for: A third generation vaccine for human visceral leishmaniasis and post kala azar dermal leishmaniasis: First-in-human trial of ChAd63-KH
Source: PLoS Negl Trop Dis. 2017 May 12;11(5):e0005527. doi: 10.1371/journal.pntd.0005527 (PMC5443534; doi:10.1371/journal.pntd.0005527)
Supplement: S1 Text — (DOCX) [file pntd.0005527.s001.docx]

**Osman et al Supplementary Materials**

**1. Supplementary Materials and Methods**

*Whole blood transcriptomics*

Total RNA was extracted from PAXgene tubes using the Qiagen PAXgene blood miRNA kit following manufacturer’s instructions. With the PAXgene Blood miRNA Kit, all RNA molecules longer than 18 nucleotides are purified. RNA Tubes were first centrifuged to pellet the samples, which were then washed with water and resuspended. After digestion with proteinase K, the samples were homogenized by centrifugation through PAXgene Shredder spin columns. Isopropanol was added to the samples to optimize binding conditions, and the samples were then centrifuged through PAXgene RNA spin columns, where total RNA >18 nucleotides (including miRNA) binds to the PAXgene silica-membrane. The bound RNA was subjected to two rounds of DNase digestion and clean-up using the Ambion Turbo DNase to remove genomic DNA contamination. Total RNA was depleted with the Globin-Zero™ Magnetic Gold Kit (Human/Mouse/Rat) from Epicentre using 1μg of starting material into the standard kit. The performance of each rRNA depletion was quality controlled using Bioanalyzer and Qubit. RNA–Seq libraries were prepared from the enriched material using the Epicentre ScriptSeq v2 RNA-Seq Library Preparation Kit and following 15 cycles of amplification, libraries were purified using AMPure XP beads. Each library was quantified using Qubit and the size distribution assessed using the Agilent 2100 Bioanalyser. These final libraries were pooled in equimolar ratios using the Qubit and Bioanalyzer data as 4 individual pools, 2 pools with 8 libraries per pool, and 2 pools with 8 libraries per pool. The quantity and quality of each pool was assessed by Bioanalyzer and subsequently by qPCR using the Illumina Library Quantification Kit from Kapa on a Roche Light Cycler LC480II according to manufacturer's instructions. Each pool of RNA Seq libraries was sequenced on one lane of the HiSeq 2500 at 2x125 bp paired-end sequencing with v4 chemistry.

*Bioinformatic analysis*

The reference genome used for alignment was the human reference genome assembly GRCh37/hg19. R1/R2 read pairs were mapped to the reference sequence using TopHat2 version 2.0.10 [1], which calls the mapper Bowtie2 version 2.1.0[2]. Paired-end mapping was carried out using default parameters except for the option to report a maximum of 1 alignment to the reference for each read, instead choosing the alignment with the best alignment score (or randomly choosing among equally high scoring alignments) (option “-g 1”). Read counts per gene were calculated using HTSeq-count (http://wwwhuber.embl.de/users/anders/HTSeq/doc/count.html). Differential gene expression (DGE) analysis was applied to the read count data for reads mapped to the human genome.

The analysis was conducted in the R environment using edgeR [3]. Initial processing and quality assessment of the sequence data was performed using an

in-house pipeline (developed by Dr Richard Gregory, MRC Centre of Genomics).

Briefly, base calling and de-multiplexing of indexed reads was performed by CASAVA

version 1.8.2 (Illumina) to produce 30 samples from the 4 lanes of sequence data, in

fastq format. The raw fastq files were trimmed to remove Illumina adapter sequences

using Cutadapt version 1.2.1[4]. The option “-O 3” was set, so the 3' end of

any reads which matched the adapter sequence over at least 3 bp was trimmed off. The

reads were further trimmed to remove low quality bases, using Sickle version 1.200 with

a minimum window quality score of 20. After trimming, reads shorter than 10 bp were

removed. If both reads from a pair passed this filter, each was included in the R1

(forward reads) or R2 (reverse reads) file. If only one of a read pair passed this filter, it

was included in the R0 (unpaired reads) file.

*Data modelling for the gene expression data*

The DGE analysis was designed as follows. The 30 high dose samples were associated with 2

levels of the factor “time”, which are v2 pre dose (T1) and v3 post dose. 15 biological

replicates were employed in this experiment. A generalised linear model (GLM) with two

model coefficients was employed to estimate and test the fold change associated v3_VS_v2 contrast. The two parameters are: intercept Mean expression of all samples v3_VS_v2 log2 Fold change of v3 against v2 contrast.

*Model fitting and variance estimation for the gene expression data*

Normalisation factors were calculated to correct for differences in size among samples, which may otherwise cause bias in differential gene expression analysis. The “TMM” (Trimmed Mean M-values) method in edgeR was applied, with default parameters. Taking a negative binomial (NB) distribution to formulate the data variation, the dispersion parameter for the distribution was estimated based on the data and model matrix using the GLM approach. Common, trended and tag-wise dispersion parameters were estimated (plotted in Figure 5). Tagwise dispersion was used for fold change estimating and significance testing.

*Testing logFC for the contrasts and detecting DE genes*

The estimated log2 Fold Change for the contrasts were tested in edgeR using a

Likelihood-Ratios (LR) test [5]. P-values associated with logFC (log2 Fold

Change) were adjusted for multiple testing using the False Discovery Rate (FDR)

approach [6]. Significantly differentially expressed genes were defined as those with an FDR-adjusted P-value < 5%. The DE genes are shown in Tables S3 and S4.

1. Kim D, Pertea G, Trapnell C, Pimentel H, Kelley R, Salzberg SL. TopHat2: accurate alignment of transcriptomes in the presence of insertions, deletions and gene fusions. Genome biology. 2013;14(4):R36. Epub 2013/04/27. doi: 10.1186/gb-2013-14-4-r36. PubMed PMID: 23618408; PubMed Central PMCID: PMC4053844.

2. Langmead B, Salzberg SL. Fast gapped-read alignment with Bowtie 2. Nature methods. 2012;9(4):357-9. Epub 2012/03/06. doi: 10.1038/nmeth.1923. PubMed PMID: 22388286; PubMed Central PMCID: PMC3322381.

3. Robinson MD, McCarthy DJ, Smyth GK. edgeR: a Bioconductor package for differential expression analysis of digital gene expression data. Bioinformatics. 2010;26(1):139-40. Epub 2009/11/17. doi: 10.1093/bioinformatics/btp616. PubMed PMID: 19910308; PubMed Central PMCID: PMC2796818.

4. Martin M. Cutadapt removes adaptor sequences from high throughput sequencing reads. EMBnetjournal. 2011;17:10-2.

5. Wilks SS. The large-sample distribution of the likelihood ratio for testing composite hypotheses. Annals of Mathematical Statistics. 1938;9:60-2.

6. Benjamini Y, Hochberg Y. Controlling the false discovery rate: a practical and powerful approach to multiple testing. Journal of The Royal Statistical Society, Series B. 1995;57:289-300.
