# Supplementary material for: A third generation vaccine for human visceral leishmaniasis and post kala azar dermal leishmaniasis: First-in-human trial of ChAd63-KH
Source: PLoS Negl Trop Dis. 2017 May 12;11(5):e0005527. doi: 10.1371/journal.pntd.0005527 (PMC5443534; doi:10.1371/journal.pntd.0005527)
Supplement: S5 Table — (PDF) [file pntd.0005527.s006.pdf]

Table S5 HLA typing of study subjects

Donor	Group	HLA Class 1	HLA Class II
1	1	A1, A2; B8, B39; Cw7	DR13, DR17; DQ2, DQ7; DR52
3	1	A1, A3; B7, B35 ;Cw4, Cw7	DR1, DR4; DQ5, DQ7; DR53
4	1	A2, A29; B44, B50; Cw6, Cw16	DR7, DR15; DQ2, DQ6; DR51, DR53
6	1	A2; B44, B51; Cw2, Cw5	DR7, DR15; DQ2, DQ6; DR51, DR53
10	1	A2, A68; B7, B71; Cw7	R12, DR15; DQ6, DQ7; DR51, DR52
11	2	A2, A3; B18, B60 ;Cw7, Cw10	DR11, DR13; DQ6, DQ7; DR52
12	2	A1,A3;B8,B18;CW5,CW7	DR17; DQ2 ;DR52
15	2	A3,A24;B7,B27;CW2,CW7	DR4, DR15; DQ6, DQ8; DR51, DR53
16	2	A1,A24;B39,B64;CW7,CW6	DR7, DR13; DQ2, DQ6; DR52, DR53
17	2	A2;B7,B35;W4,CW7	DR1, DR15; DQ5, DQ6; DR51
18	2	A3,A29;B7,B44;CW7,CW16	DR7, DR15; DQ2, DQ6; DR51, DR53
19	2	A11,A24;B7,B44,CW5,CW7	DR4, DR11; DQ7; DR52, DR53
21	2	A1,A24;B18,B39;CW5,CW7	DR1, DR17; DQ2, DQ5; DR52
22	2	A1,A3,B8,B44;CW2,CW7	DR1, DR17; DQ2, DQ5; DR52
23	2	A1,A24;B35,B55;CW4,CW9	DR103, DR13; DQ5, DQ6; DR52
24	2	A2,A24;B51,B57;CW6,CW7	DR4, DR7; DQ7, DQ9; DR53
26	2	A3,A29;B35,B44;CW4,CW16	DR1, DR7; DQ2, DQ5; DR53
27	2	A2,A24;B44,B55;CW5,CW9	DR4, DR13; DQ6, DQ7; DR52, DR53
28	2	A1,A3;B8,B35;CW7,CW12	DR4, DR11; DQ7, DQ8; DR52, DR53
32	2	A3,B7;B65;CW7,CW8	DR7, DR15; DQ2, DQ6; DR51, DR53
