# Supplementary figures and images for: A third generation vaccine for human visceral leishmaniasis and post kala azar dermal leishmaniasis: First-in-human trial of ChAd63-KH
Source: PLoS Negl Trop Dis. 2017 May 12;11(5):e0005527. doi: 10.1371/journal.pntd.0005527 (PMC5443534; doi:10.1371/journal.pntd.0005527)

Figure S1

A

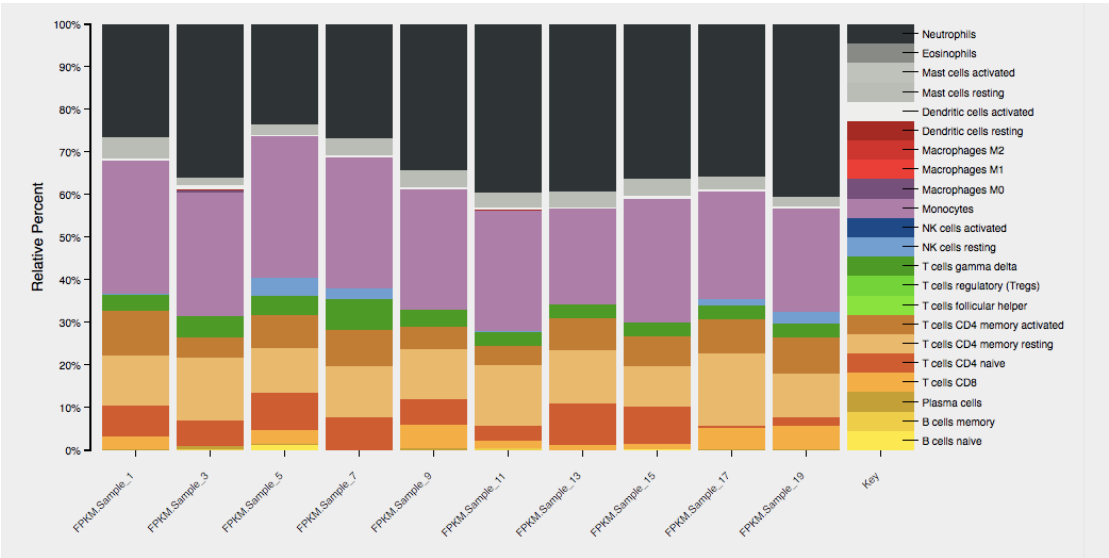

B

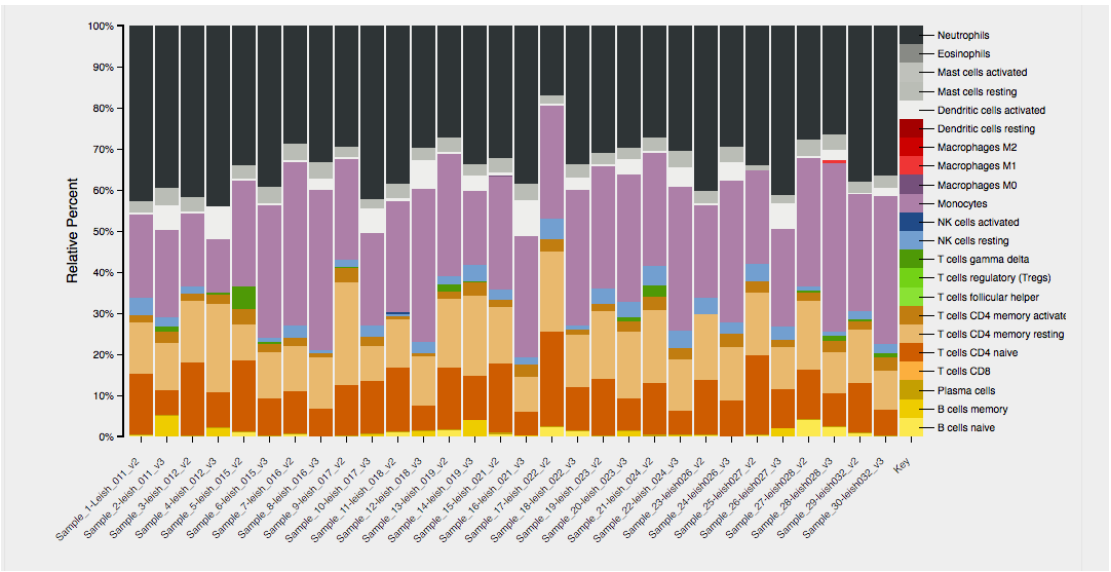

C

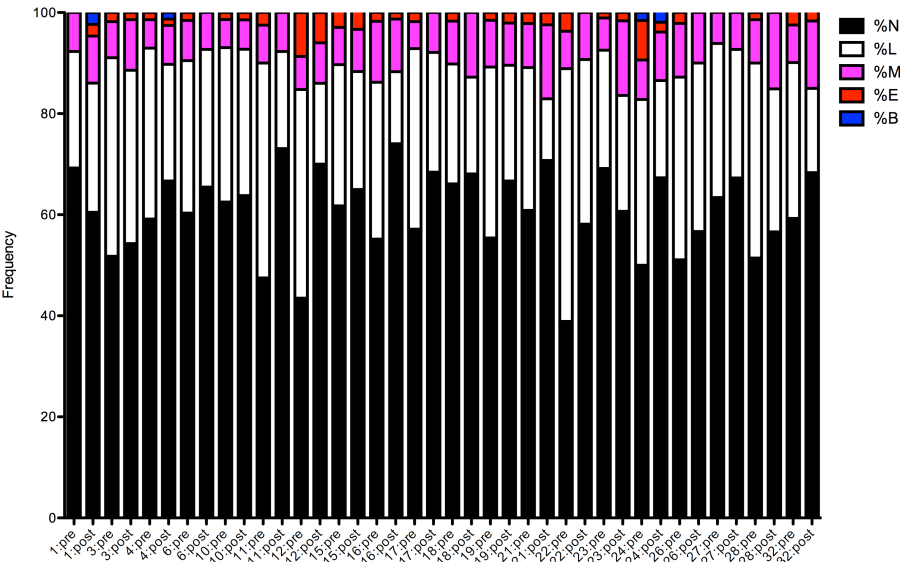

Supplement: S1 Fig — A and B. High resolution blood composition was inferred by deconvolution of whole blood RNA-Seq data using CIBERSORT. Data are shown for each low dose (A) and high dose (B) subject pre- and post-vaccination. C. Differential blood counts obtained by routine clinical hematology for all subjects pre and post vaccination. (PDF) [file pntd.0005527.s007.pdf]

Figure S2

A

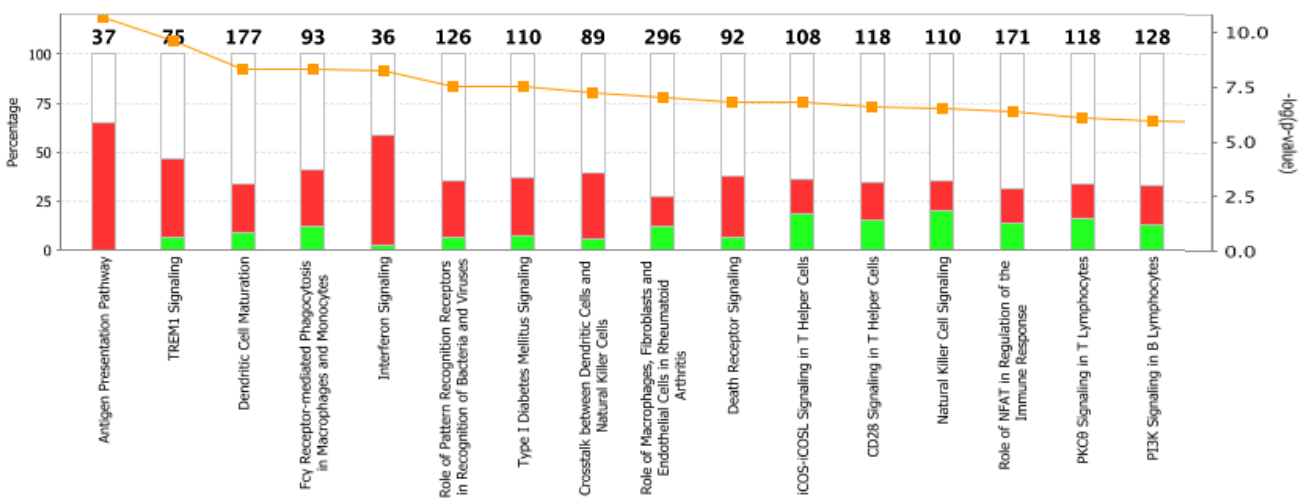

B

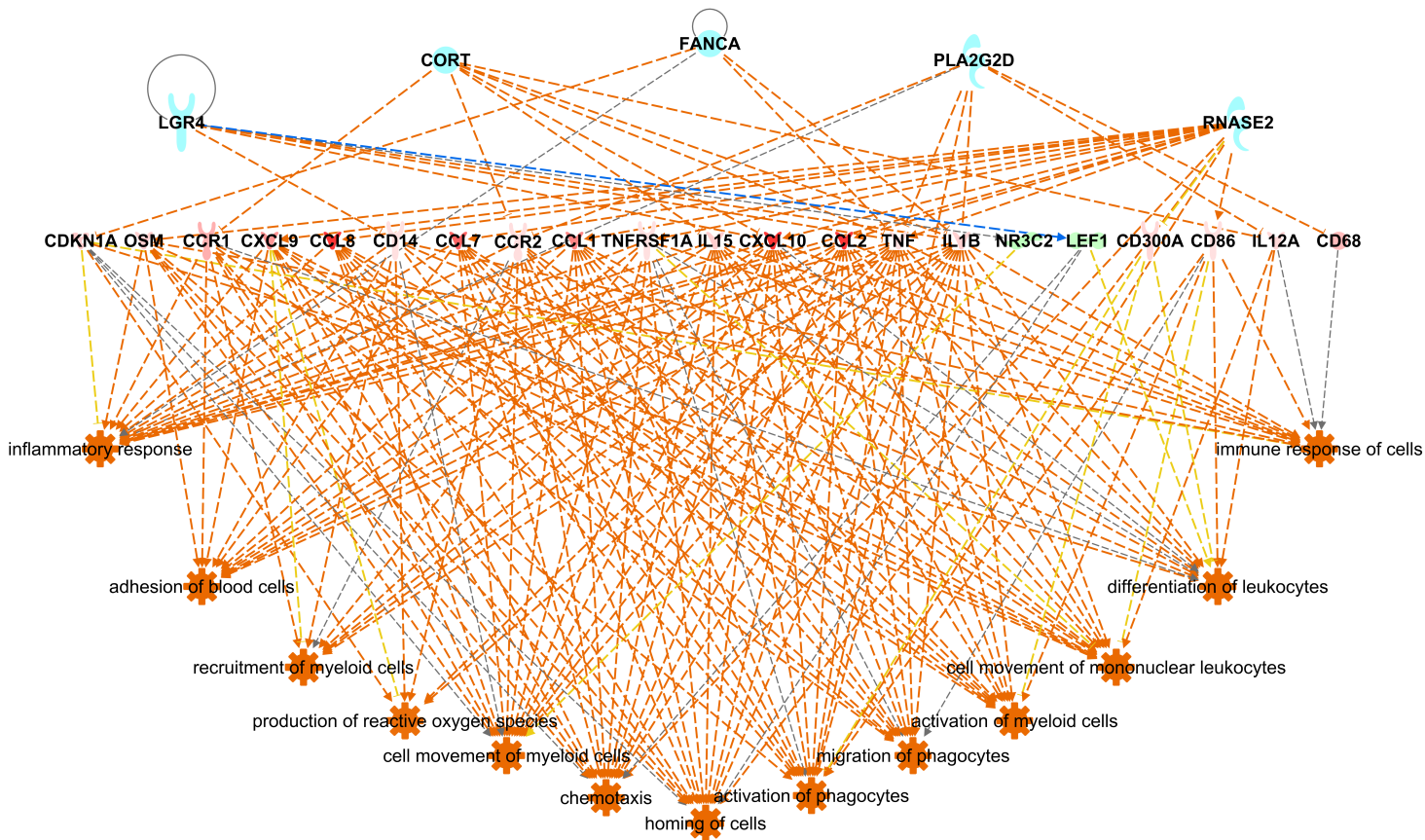

Supplement: S2 Fig — A. Top 16 enriched IPA canonical pathways. Bars (left axis) indicate percentage of genes per pathway up-regulated (red) or down-regulated (green). Line graph (right axis) indicates log10 probability (P value) vs. randomly selected gene group of same size. Numbers above bars indicate the number of genes included each pathway. B. IPA-derived regulator map for monocyte / macrophage activation. (PDF) [file pntd.0005527.s008.pdf]

Figure S3

A

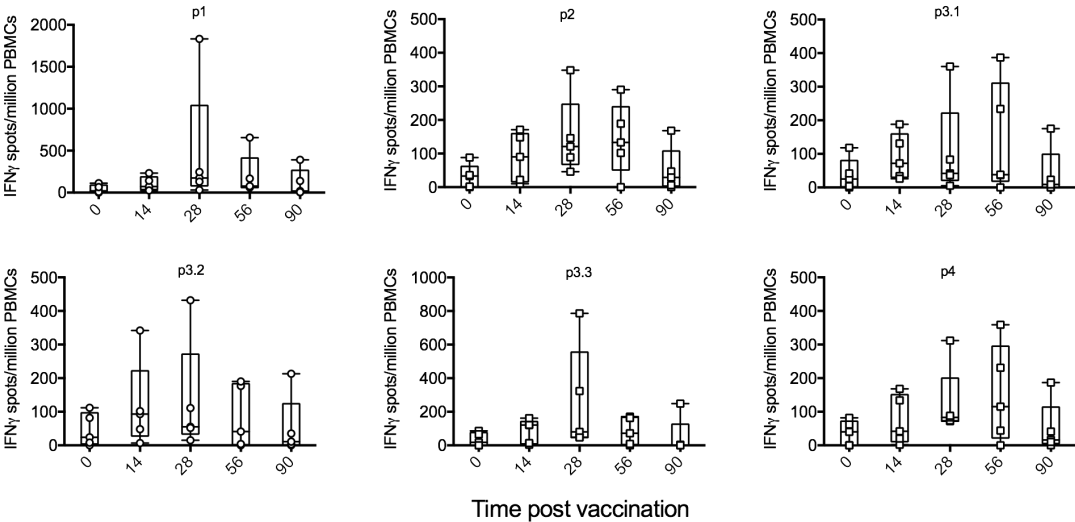

B

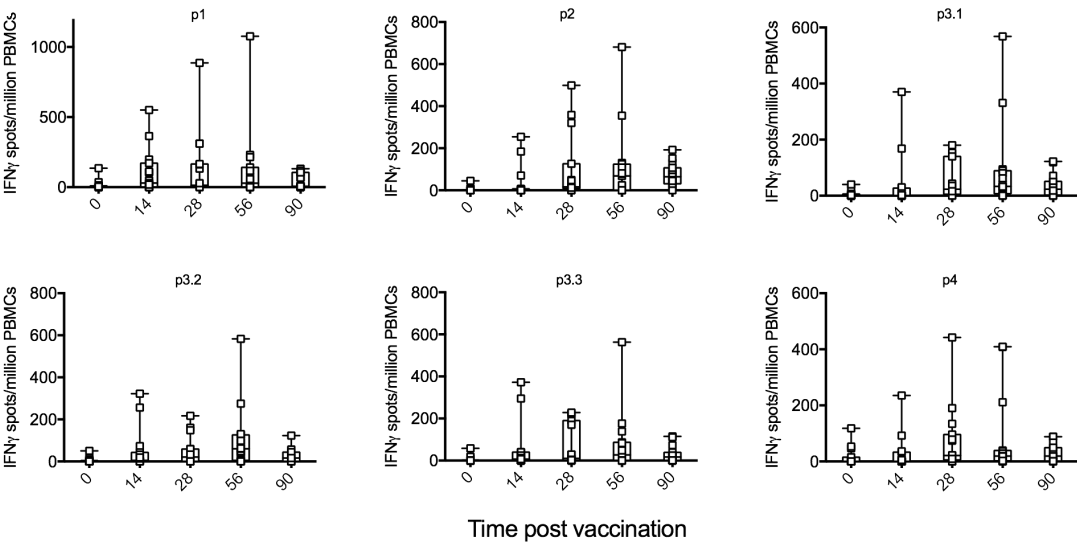

C

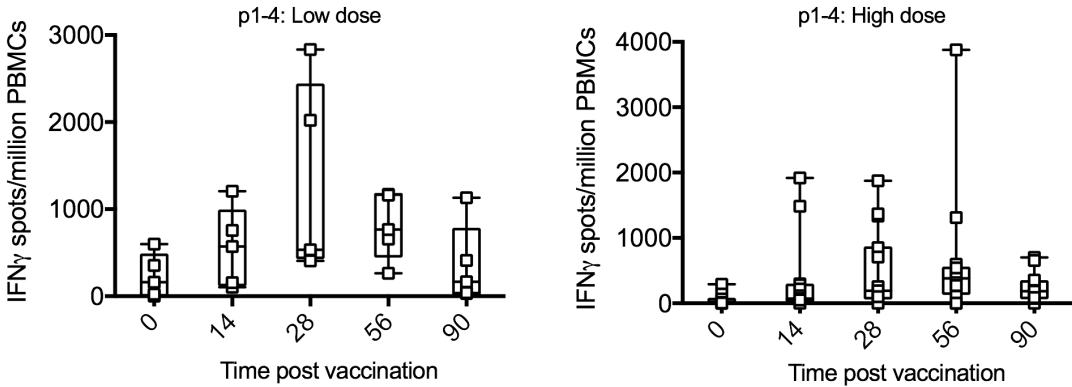

Supplement: S3 Fig — A-C. IFNγ response determined by ELISPOT before and at the indicated times following vaccination of low dose (A and C, left panel) and high dose (B and C, right panel) subjects to individual peptide pools spanning the KH antigen (A, B). The summed response is also shown (C). Data are shown as box and whisper plots. (PDF) [file pntd.0005527.s009.pdf]

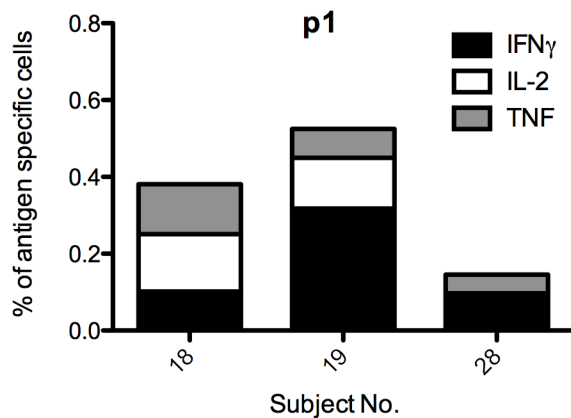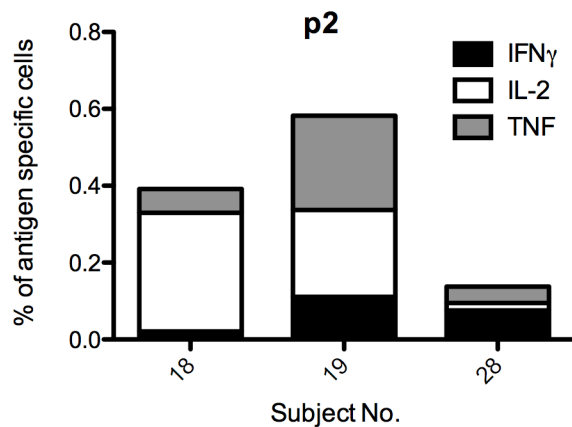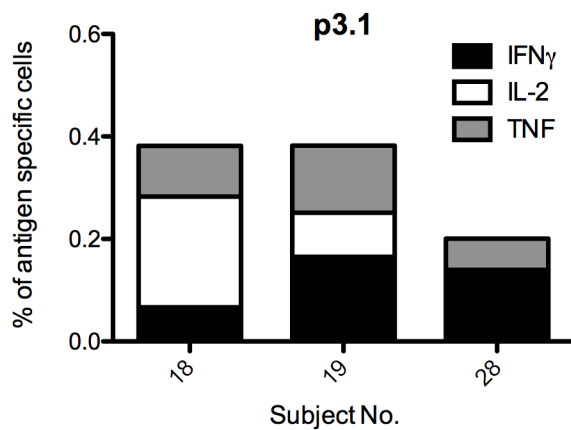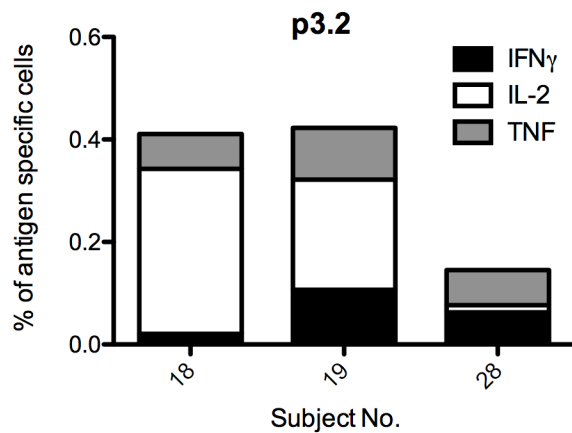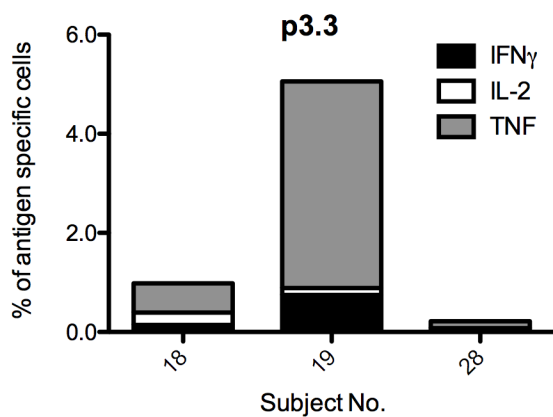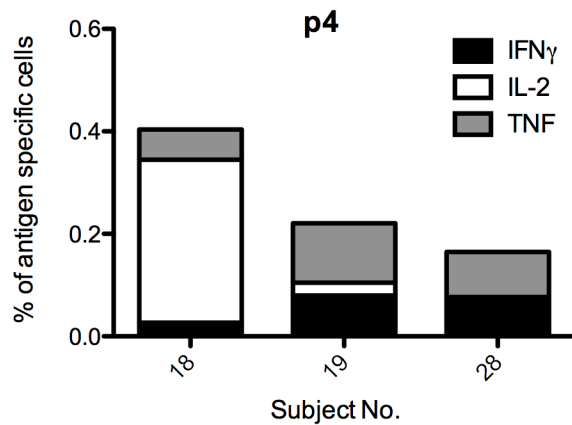

Supplement: S4 Fig — IFNγ (black bars), TNF (grey bars) and IL-2 (white bars) were measured by ICS in CD8+ T cells at day 28 post-vaccination for ELISPOT non-responder subjects 18, 19 and 28. Data represent mean frequency of antigen-specific T cells producing each cytokine in response to peptide pools spanning the KH antigen (p1, p2, p3.1, p3.2, p3.3, p4). (PDF) [file pntd.0005527.s010.pdf]
